# Life scientists improve QSP model quality and impact

**DOI:** 10.3389/fphar.2024.1392747

**Published:** 2024-07-02

**Authors:** Katherine Kudrycki, Christina Friedrich, Mike Reed, Rebecca A. Baillie

**Affiliations:** Rosa & Co LLC, San Carlos, CA, United States

**Keywords:** quantitative systems pharmacology, pharmacometrics, modeling, drug development, biologist

## 1 Introduction

Quantitative systems pharmacology (QSP) informs all stages of drug development by integrating data and knowledge about physiology, pathophysiology, and pharmacology into mechanistic models (Azer et al., 2021; Sher et al., 2022). QSP models are used to improve knowledge about a compound’s mechanism of action and optimize dosing, identify valuable biomarkers, and predict efficacy across patient populations (Cucurull-Sanchez, 2024). QSP models represent biological systems, but paradoxically, modeling teams rarely include full-time life scientists with an advanced degree (MS, PhD, MD) in biochemistry, physiology, or medicine. A 2018 industry-wide survey of pharmaceutical companies revealed that fewer than one in 10 QSP team members identified as biologists; most QSP modelers have backgrounds in Pharmaceutical Sciences, Engineering, and Computational Biology/Bioinformatics (Nijsen et al., 2018). Job descriptions and education requirements for QSP modelers often require software development skills and training in clinical pharmacology, pharmacokinetics, or applied mathematics but require little or no biological sciences background (Androulakis, 2022; Zhang et al., 2022b; Gallo, 2022). In our capacity as consultants interacting with modeling groups in pharmaceutical companies, we have observed that it is exceedingly rare for life scientists to be core members of modeling teams despite some recommendations (Leil and Bertz, 2014; Aghamiri et al., 2022). However, a life scientist’s years of training and experience are critical for providing biological knowledge, assessing data quality, and interpreting results, which may be challenging for non-biologists. Having a life scientist participate in all phases of QSP modeling improves model development and outcomes.

## 2 Life scientists provide essential input for QSP model design

The value of QSP research results depends on the biological data and knowledge used to develop and qualify the model. No amount of mathematical or engineering expertise can compensate for poor data inputs or inappropriate data interpretation. Therefore, the judgment of a life scientist who understands the disease pathophysiology and data sources is critical for defining the model scope and data curation. They can identify and extract the most reliable and relevant data and improve the quality, applicability, and use of data during the modeling process. However, to fully utilize the expertise of life scientists, they should be embedded in the modeling team or contribute to modeling on a dedicated basis rather than sporadically during the model development process (Leil and Bertz, 2014). Because these life scientists are familiar with the biology supporting the drug development question, they are more able to identify and quantify the range of biological species, parameters, behaviors, and qualification tests that need to be included in the modeling. This knowledge allows them to define better testing criteria for the model, propose hypotheses and alternative pathways or data for knowledge gaps, and provide meaningful and biologically correct data for the development and qualification of virtual patients and virtual populations. This disciplined, scientific approach is helpful at all stages of model development and application to ensure the relevance of the model to the biological question at hand.

The argument may be made that a skilled modeling team without a life scientist could also select the appropriate model scope, parameters, and qualification experiments through diligent research and data collection. However, a life scientist’s training and experience under real-world project conditions are better adapted to these tasks. They can provide significant advantages in project time and model relevance. In addition, while mathematical and engineering concepts are often readily transferrable across models, the biological scope and application of the model can vary widely depending on the project; therefore, having a trained life scientist support the modeling can be more efficient. Having a life scientist support the modeling allows the modelers to quickly acquire a more nuanced understanding of the relevant biology because they collaborate with experts.

A potentially perilous scenario is when a modeling team embarks on modeling an unfamiliar pathophysiology. Internet searches provide background information, from Wikipedia to research reviews; however, these may include too much or too little information to develop an appropriate model architecture. Building an overly complex model may obscure rather than clarify critical biological behaviors due to model redundancies and delay the delivery of essential results, increasing their cost and reducing their utility. Often, review articles list all the possible biological components and connections, emphasizing elements that may have minor physiological roles simply because they are new or novel. Alternatively, review articles may omit quantitative data critical to represent the biological aspect of the model. Review articles and websites may lack citations for original data or be biased toward the author’s opinions and hypotheses (Brown et al., 2017). The embedded life-science expert is trained to spot these limitations and recognize scientific bias. Life scientists brought into a modeling project to provide a single expert opinion or scientists borrowed from other parts of the organization to contribute to modeling in their spare time would not be able to perform these tasks and may overlook critical connections.

### 2.1 “…but this molecule is important! we must include it!”

Scientific knowledge is constantly changing. What we knew and treated as dogma 10 years ago may get upended by recent discoveries. Based on the amount of scientific “chatter” in the literature, a modeling team may conclude that the new hot discovery is important enough to be included in the model. Suppose the life-science expert serves only on a consulting basis. In that case, the team may get scientific buy-in from an expert who is equally mesmerized by the new exciting discovery and proceed with inclusion. But is this decision correct? For instance, the newly described “molecule of the year,” even if it is relevant to the pathophysiology represented in the model, may not aid in answering the desired research questions and, thus, should be considered out of scope. The functions attributed to the new molecule may be redundant with other previously described molecular pathways. Finally, there may not be sufficient data to support inclusion in the model. A life-science expert embedded in the team will understand the ramifications of expanding the scope. They can quickly advise the team on whether including the new component would enhance the model and help answer research questions or if it would increase uncertainty and reduce the usefulness of predictions.

Thus, the importance of life scientists’ expertise is evident during the scoping stage of model development because life scientists research, analyze, and synthesize the data to construct the model architecture. In addition, they help avoid pitfalls related to the model’s size and scope, and they can determine whether individual components are important enough to be included in the model.

### 2.2 Without a life scientist, modelers may gain false confidence during model qualification

Without input from an embedded life scientist, modelers risk building a model that shows good agreement with previously measured outcomes but has biologically unrealistic parameter values or behaviors that would be clear to a life scientist. For example, some seemingly disparate physiological pathways or reactions are coordinately regulated and vary in tandem. This coordinated regulation may not be obvious to someone without a life scientist background. It would not be appropriate to vary these pathways independently either as part of a sensitivity analysis or in the development of a virtual population. While it might be possible to create virtual patients whose individual measurements are all in the normal range, the relationships between the measures would not make biological sense.

Teams focusing more on modeling techniques that neglect biological relevance are also at risk of sacrificing the quality of their results. Best practices emphasize that QSP models should integrate pharmacology and biology (Helmlinger et al., 2019). A life scientist embedded in the modeling team can help curate the biological data, define validation criteria, and bring their broader scientific background and judgment to assessing the model and virtual patients. They can apply qualitative and quantitative calibration criteria, for example, ensuring that results are consistent with evidence from related therapeutic areas or animal models that may not be suitable as quantitative calibration data (Singh et al., 2023). This work is particularly crucial in areas with little direct data for calibration and validation, where inferences and judgment must be used to make reasonable assumptions.

Artificial intelligence, machine learning, and large language models are good tools for QSP modeling that function best with large amounts of data (Azer et al., 2021; Zhang et al., 2022a; Terranova et al., 2024). These tools still require someone to evaluate, interpret, and curate the input data and output results. The usefulness of these tools is reduced for diseases and biological areas with limited direct data. These are the same areas where QSP models are the most helpful and a life scientist’s input the most valuable (Bai et al., 2024). In the future, these tools may be more autonomous, but for now, they should be used with appropriate input from both engineering and life science team members.

### 2.3 Life scientists can interpret simulation results to enable actionable results

In a project’s research phase, the modeling team needs sufficient expertise to interpret the data: Do the results make physiological sense? Are they compatible with the existing biological data? Life scientists are best positioned to explain whether and why the results are reasonable to the broader scientific organization. If the simulation results are unexpected, the life scientist can determine whether formulating and testing alternative hypotheses in the existing model is appropriate or whether different biology or additional research is required.

## 3 Discussion

QSP is the mathematical modeling of biological systems and their interactions with pharmacological interventions. Because life scientists have specific expertise in disease pathology and physiology, they are critical to the success of QSP modeling teams. Life scientists should be part of the core modeling team to contribute meaningfully to each project stage (Table 1).

**TABLE 1:** Value added by a life scientist.

QSP task	Life scientist value added
Model design	Accurate representation of pathophysiology, drug target, and pathway interactions with the target. Better fit to purpose. Reasonable assumptions
Data selection, analysis, publication vetting	Improved data quality, consistency, and applicability
Model testing and calibration	Appropriate model constraints, improved submodule calibration
Virtual patient development	Model/VP behavior is consistent with patients in clinical trials. Parameters defining the VPs have realistic values
Communication	Can explain the model and results to clinicians and stakeholders
Corporate intellectual property	Appropriate use and reuse of data and knowledge transfer across multiple modeling projects

Life scientists with limited input are less familiar with the model, have less time to focus on modeling concepts and data analyses, are less able to transfer knowledge across projects, and are less familiar with the assumptions and decisions that result in modeling constraints. Being part of the core team allows life scientists to spend adequate time identifying and analyzing the data and assessing the model and its simulated outputs. Core-team life scientists can raise critical questions and considerations during model development (particularly those that may not occur to a modeler) and are in a position to recognize when model behavior is consistent with relevant constraints. Life scientists who learn about mathematical and modeling concepts by participating at all stages of a project can apply data and the resulting knowledge across multiple future projects.

Educational programs and workshops could include training for life scientists to better support QSP teams. Recommended coursework might include communication, operations research, dynamics, mathematical modeling, and programming or use of the appropriate software. The goal is not to make the life scientist an expert modeler, but rather to improve understanding and communication of the modeling and biology.

Life scientists on the core team also help communicate results in a way relevant to the project’s stakeholders. Having dedicated life scientists on a team leads to the more efficient building and qualification of the model and the improved interpretation of results. Thus, this inclusion helps ensure that a model is biologically sound and that modeling results are meaningful to the stakeholders.
